# Properties of cortical axons for energy efficient cortical action potentials

**DOI:** 10.1186/1471-2202-13-S1-P6

**Published:** 2012-07-16

**Authors:** Yuguo Yu

**Affiliations:** 1 Center for Computational Systems Biology, Fudan University, Shanghai, 200433, China; 2 School of Life Sciences, Fudan University, Shanghai, 200433, China

## 

**Figure 1:**
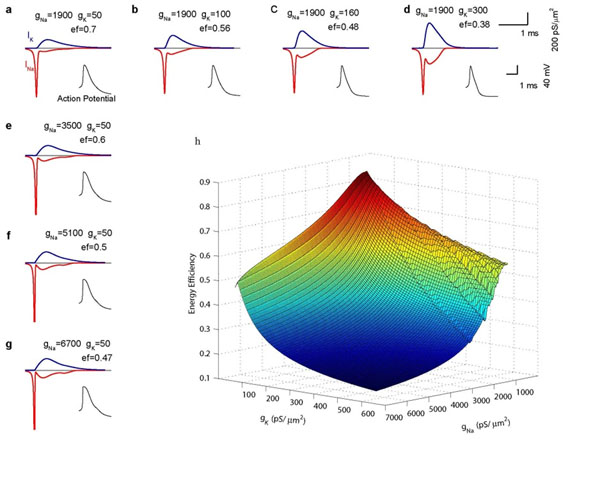
Spike energy efficiency is dominated by values of Na+ and K+ channel conductance.

Brains are extremely energy demanding. Recent studies [1] have demonstrated that AP generated in mammalian neurons are more energy efficient than squid giant axon [2]. In an companion study, we have showed that a warm body temperature is a critical factor responsible for the high efficient cortical action potentials in endotherm brain neurons than those in ectotherm neurons. In this study, we investigate that besides warm body temperature, what are the principal factors affecting energy cost in action potential generation. Since the energy cost is directly related to Na+ and K+ channels, we build up a cortical neuronal model based on Hodgkin-Huxley theory [3] and investigate the effect of changes in Na+ and K+ distribution density on the axon energetics. Figure 1 shows that there exist a pair of optimal Na+ and K+ conductance values, leading to the maximal energy efficiency. Below the level of the optimal Na+ conductance, the action potential could not be generated, while above this level, the energy cost will increase gradually. For a fixed Na+ conductance, a lower K+ conductance results in an unstable spiking dynamics, and a higher K+ conductance results in a high energy cost. Therefore, once a neuron developed a certain amount of Na+ channel density, there exist an optimal K+ channel density, where the spike dynamics is stable while the energy cost is minimized.

Then we studied the energy efficiency for action potential initiation and propagation in unmyelinated and myelinated axons. Comparing to a point model, Na+ entry ratio (SER) increases for action potential initiation in the unmyelinated axon. This is because that an additional amount of I_Na_ is required to flow into neighborhood axon compartment when AP is propagated on the axon. This axial Na+ current results in a rapid rising phase of membrane potential, triggering a large amount of local Na+ influx for local action potential generation. Therefore, this axial Na+ current saves energy for action potential generation in the neighborhood compartment during AP propagation. Compared to the unmyelinated axon, spike propagation in a myelinated axon saves energy extensively for AP propagation.

In summing, the energy cost for action potential initiation and propagation in cortical axons is not only ionic channel dependent, but also axonal property dependent.
